# Latent class analysis of psychotic-affective disorders with data-driven plasma proteomics

**DOI:** 10.1038/s41398-023-02321-9

**Published:** 2023-02-06

**Authors:** Sang Jin Rhee, Dongyoon Shin, Daun Shin, Yoojin Song, Eun-Jeong Joo, Hee Yeon Jung, Sungwon Roh, Sang-Hyuk Lee, Hyeyoung Kim, Minji Bang, Kyu Young Lee, Se Hyun Kim, Minah Kim, Jihyeon Lee, Jaenyeon Kim, Yeongshin Kim, Jun Soo Kwon, Kyooseob Ha, Youngsoo Kim, Yong Min Ahn

**Affiliations:** 1 Biomedical Research Institute, Seoul National University Hospital, Seoul, Republic of Korea; 2 Department of Biomedical Sciences, Seoul National University College of Medicine, Seoul, Republic of Korea; 3 Department of Psychiatry, Seoul National University College of Medicine, Seoul, Republic of Korea; 4 Department of Neuropsychiatry, Seoul National University Hospital, Seoul, Republic of Korea; 5 Department of Neuropsychiatry, School of Medicine, Eulji University, Daejeon, Republic of Korea; 6 Department of Psychiatry, Uijeongbu Eulji Medical Center, Eulji University, Uijeongbu, Republic of Korea; 7 Department of Psychiatry, SMG-SNU Boramae Medical Center, Seoul, Republic of Korea; 8 Institute of Human Behavioral Medicine, Seoul National University Medical Research Center, Seoul, Republic of Korea; 9 Department of Psychiatry, Hanyang University Hospital and Hanyang University College of Medicine, Seoul, Republic of Korea; 10 Department of Psychiatry, CHA Bundang Medical Center, CHA University School of Medicine, Seongnam, Republic of Korea; 11 Department of Psychiatry, Inha University Hospital, Incheon, Republic of Korea; 12 Department of Psychiatry, Nowon Eulji University Hospital, Seoul, Republic of Korea; 13 Institute of Medical and Biological Engineering Medical Research Center, Seoul National University College of Medicine, Seoul, Republic of Korea

**Keywords:** Molecular neuroscience, Physiology

## Abstract

Data-driven approaches to subtype transdiagnostic samples are important for understanding heterogeneity within disorders and overlap between disorders. Thus, this study was conducted to determine whether plasma proteomics-based clustering could subtype patients with transdiagnostic psychotic-affective disorder diagnoses. The study population included 504 patients with schizophrenia, bipolar disorder, and major depressive disorder and 160 healthy controls, aged 19 to 65 years. Multiple reaction monitoring was performed using plasma samples from each individual. Pathologic peptides were determined by linear regression between patients and healthy controls. Latent class analysis was conducted in patients after peptide values were stratified by sex and divided into tertile values. Significant demographic and clinical characteristics were determined for the latent clusters. The latent class analysis was repeated when healthy controls were included. Twelve peptides were significantly different between the patients and healthy controls after controlling for significant covariates. Latent class analysis based on these peptides after stratification by sex revealed two distinct classes of patients. The negative symptom factor of the Brief Psychiatric Rating Scale was significantly different between the classes (*t* = −2.070, *p* = 0.039). When healthy controls were included, two latent classes were identified, and the negative symptom factor of the Brief Psychiatric Rating Scale was still significant (*t* = −2.372, *p* = 0.018). In conclusion, negative symptoms should be considered a significant biological aspect for understanding the heterogeneity and overlap of psychotic-affective disorders.

## Introduction

Psychiatric disorders, including schizophrenia (SCZ), bipolar disorder (BD), and major depressive disorder (MDD), are known to exhibit within-disorder heterogeneity and between-disorder overlap [1]. This is probably due to the diagnostic procedure for psychiatric disorders, as they are based on subjective symptoms and behavioral observations, with a lack of biological validity. Thus, to discover a more homogenous biological subgroup, recent studies have focused on identifying subtypes using data-driven approaches [2]. Finding a biological subtype in a transdiagnostic sample can contribute to deepening our understanding of the pathophysiology and the heterogeneity and overlap between disorders. Research projects, such as the Roadmap for Mental Health Research in Europe (ROAMER) [3] and the National Institute of Mental Health (NIMH) Research Domain Criteria (RDoC) [4], have emphasized the need for these approaches.

Focusing on proteomics data using a data-driven approach, efforts have been made to discover subtypes of depression [5, 6]. A previous study using the Netherlands Study of Depression and Anxiety (NESDA), clustered patients with depression and anxiety based on biological features, including blood proteomics, and discovered three classes based on metabolic health [6]. However, to the best of our knowledge, no study has focused on proteomics-based cluster analysis in a transdiagnostic psychotic-affective disorder spectrum to date. This study was based on a previous study in which we differentiated SCZ, BD, and MDD using plasma proteins [7]. In this study, we aimed to identify clusters based on peripheral plasma proteomic data. Demographic and clinical characteristics were compared across the identified classes to understand the differences between them.

## Materials and methods

### Clinical samples

The study population comprised 515 patients (171 SCZ, 170 BD, and 174 MDD) and 160 healthy controls (HC) aged 19–65 years who were enrolled between August 2018 and December 2020. Statistical analysis was performed, excluding 11 patients who had missing covariate values. Patients were enrolled from Seoul National University Hospital; Nowon Eulji Medical Center, Eulji University; Seoul Metropolitan Government, Seoul National University Boramae Medical Center; Hanyang University Hospital; Inha University Hospital; and Cha University Bundang Medical Center. HC were recruited from Seoul National University Hospital via advertisements. The diagnosis was based on the Diagnostic and Statistical Manual of Mental Disorders, Fifth edition (DSM-5), and confirmed using the Mini-International Neuropsychiatric Interview (MINI). HC had no psychiatric diagnosis based on the MINI and no known psychiatric family history among first- and second-degree relatives.

Patients and HC were excluded based on the following criteria: use of any anti-inflammatory analgesics (including nonsteroidal anti-inflammatory drugs and steroids, with the exception of acetaminophen) for the previous 2 weeks before participation; a history of neuromodulation or neurosurgery; central nervous system (CNS) diseases (including epilepsy, meningitis, Parkinson’s disease, and stroke); cancer; tuberculosis; current lactation or pregnancy; a history of substance abuse other than alcohol, caffeine, and nicotine; intensive psychotherapy for the previous 2 months before participation; predicted intellectual disability; and difficulty in interpreting Korean. Current psychotropic medication use was not an exclusion criterion. Most studies were based on previous reports on the association between these conditions and altered protein expression [7]. Those who had recently received neuromodulation and psychotherapy were excluded to confine the treatment effects to psychotropic medications.

Plasma samples were collected in a 6-mL ethylenediaminetetraacetic acid (EDTA) tube (ref 367863; Becton, Dickinson and Company, Trenton, NJ) and centrifuged at 1100–1300×*g* for 10–15 min at room temperature or 4 °C. The supernatant was collected and stored in an Eppendorf tube at ≤−70 °C.

The authors assert that all procedures contributing to this work comply with the ethical standards of the relevant national and institutional committees on human experimentation and the Helsinki Declaration of 1975, as revised in 2008. All procedures involving human subjects/patients were approved by the Institutional Review Boards of Seoul National University Hospital (IRB No. 1806-1065-951) and all other participating hospitals. Written informed consent was obtained from each participant after the procedure was fully explained.

### Demographics and clinical features

The demographics considered were age, sex, body mass index (BMI), blood collection time, fasting time, current alcohol use, current exercise status, and current smoking status. Age and BMI were analyzed as continuous variables, and sex (men/women), blood collection time (AM, PM), fasting time (<8, ≥8 h), current alcohol use (yes/no), current exercise status (yes/no), and current smoking status (yes/no) were analyzed as dichotomous variables. Current alcohol use was defined as having at least one drink once per week. Current exercise status was defined using the World Health Organization’s recommendation of moderate-intensity physical activity for at least 30 min once per week [8].

For patients, medication use was analyzed as a dichotomous variable for antipsychotics, lithium/anticonvulsants, antidepressants, and benzodiazepines/hypnotics. The chronicity of the disease or medication was assessed using the continuous parameters of the duration from first onset (years) and duration from first medication (years).

The primary symptoms considered for analysis were clinician rater scales. Symptom severity was assessed using the Brief Psychiatric Rating Scale (BPRS) [9], Young Mania Rating Scale [10], Montgomery–Asberg Depression Rating Scale (MADRS) [11], and Hamilton Anxiety Scale [12]. Four factors of the 24-item BRPS were also considered based on a previous study [13].

The following scales were used for subjective reports. The Symptom Checklist-90-Revised [14] was considered for subjective symptoms. A brief form of the World Health Organization Quality of Life Assessment Instrument [15], Childhood Trauma Questionnaire [16], short form of the Wender Utah Rating Scale [17], Composite Scale of Morningness [18], and Seasonal Pattern Assessment Questionnaire [19] were also used.

### Plasma proteomic quantification

More specific methods for targeted proteomic analysis have been described previously [7], and are described in Supplementary methods. For each plasma sample, the six most abundant proteins were depleted using a MARS-6 column (Agilent Technologies, Santa Clara, CA, USA). A total of 100 μg of protein was reduced with 0.2% RapiGest and 20 mM dithiothreitol at 60 °C for 1 h and alkylated with 100 mM iodoacetamide in the dark at room temperature for 30 min. The samples were then digested with trypsin solution at 37 °C for 4 h. Digestion was completed by adding 10% formic acid. The sample was centrifuged at 4 °C for 1 h, and the supernatants were spiked with crude stable isotope-labeled internal standard (SIS) peptides, in which a C-terminal lysine or arginine was heavy isotope-labeled (^13^C_6_^15^N_2_ or ^13^C_6_^15^N_4_) [purity >70%].

Liquid chromatography–multiple reaction monitoring–mass spectrometry was performed using a 1260 Infinity HPLC system coupled to an Agilent 6490 triple quadrupole MS (Agilent Technologies, Santa Clara, CA, USA). For each digested sample, 40 μL was injected into a guard column (2.1 × 15.0 mm, 1.8 μm, 80 Å) (Agilent Technologies, Santa Clara, CA, USA), and online desalting was conducted in 3% solvent B (formic acid/acetonitrile (v/v)) at 50 μL/min for 10 min. After the valve position was switched, the sample was transferred to the analytical column (0.5 × 35.0 mm, 3.5 μm, 80 Å) (Agilent Technologies, Santa Clara, CA, USA) in 3% solvent B at 40 μL/min for 5 min. The bound peptides were separated on the column and eluted with a linear gradient of 3–35% solvent B at 40 μL/min for 50 min.

Mass spectra were generated in positive ion mode. The collision energy was optimized by adding the intensities of the individual transitions that resulted in the largest peak areas. Only SIS peptides corresponding to the 642 target peptides were initially analyzed to evaluate their retention times. The retention times were then compared with those of endogenous target peptides by analyzing the matrix of endogenous peptides with SIS peptides of the targets and a heavy β-galactosidase peptide [purity >99%]. Subsequently, the final targets were quantified in the individual blood samples.

The raw data from the liquid chromatography–multiple reaction monitoring–mass spectrometry analysis were processed using Skyline (version 19.1.0) (MacCoss Lab, Seattle, WA, USA). Peptide quantification was calculated with the peak area ratio (PAR), which is the ratio of the endogenous to SIS peptide peak area. From the 642 target peptides, 54 unstable peptides with low intensities (intensity <1000), unequal retention times between light and heavy peptides, and skewed peaks were excluded. Subsequently, the final PAR values of 588 target peptides across 675 samples were normalized by the area of the heavy β-galactosidase peptide to reduce technical variability.

### Demographic differences between patients and HC

After excluding 11 patients with missing covariates, demographic differences were compared between patients with psychosis-affective disorders and HC. Categorical data were analyzed using the chi-square test, and continuous data were analyzed using independent *t*-tests.

### Determination of peptide markers differentiating patients and HC

As from our previous study, 588 stable peptide markers were found to be eligible as proteomic candidates [7]. Log2 transformation followed by batch correction for sample preparation batches using the Combat algorithm was performed using the R package proBatch [20]. Peptides with PAR values ≤0.01 or ≥100 for at least 5% of the total study population were initially excluded, in line with our previous study [7]. First, peptide markers were chosen when they were statistically significant in univariate analysis with the peptide values as the dependent variable and patients versus HC as the dichotomous independent variable. Next, peptides that were still significant when controlling for age, sex, BMI, blood collection time, fasting time, alcohol use, exercise, and smoking behavior were selected. Additional control of hospital type was conducted, and the peptides that were still statistically significant were selected as initial candidates. To eliminate any residual hospital batch effects for patient clustering, peptides were further excluded when they were statistically different between at least two hospital types after post hoc analysis (Tukey’s method) of analysis of variance (ANOVA), which was conducted in patients only.

### Latent class analysis

As there are no definite norms for these peptides, each peptide was divided into three groups, based on the first and second tertiles, and was labeled as low (−1), medium (0), and high (1), in line with a previous study [6]. The procedure was stratified by sex, as it is known to affect certain markers [21]. Latent class analysis (LCA) was conducted, with the start values increased to 2000 random sets of starting values for the initial stage and 50 for the optimization stage, and the number of iterations increased to 50. The analysis started with one class, as additional classes were added, the optimal number of latent classes was confirmed by comparing the Akaike information criteria, Bayesian information criterion (BIC), sample-size adjusted BIC (ssaBIC), entropy, Lo–Mendell–Rubin likelihood ratio, and bootstrapped likelihood ratio test. Then, the study participants were assigned to each class based on posterior class probability.

After LCA, the demographic and clinical characteristics were compared between the latent classes. Categorical and continuous data were analyzed using the chi-square test and independent t-test, respectively. Additionally, a LCA was performed, including HC. In the additional analysis, as HC differed between classes, it was controlled as a covariate when performing linear regression to compare latent classes.

### Bioinformatics analysis

The peptides that were significantly different between clusters were subjected to ingenuity pathway analysis (QIAGEN, Hilden, Germany) for network analysis. The *p* values of the chi-squared test between latent classes were subjected when performing ingenuity pathway analysis. Subsequently, the diseases/functions and canonical pathways associated with the network were predicted using Fisher’s exact test. Among the top ten diseases/functions and canonical pathways ranked according to statistical significance, diseases/functions and canonical pathways consisting of at least half of the peptides were determined.

### Statistical analysis

Statistical analyses for demographical/clinical and targeted proteomic data were performed using SPSS version 21.0 (IBM Corporation, Armonk, NY, USA) and R version 4.1.2 (https://www.R-project.org). LCA was performed using Mplus version 8.7 (Muthén & Muthén, Los Angeles, CA, USA). Statistical tests were two-tailed, and statistical significance was set at *p* value <0.05.

## Results

### Demographic differences between patients and HC

Differences in demographics between patients with psychosis-affective disorder and HC were analyzed. The patients had higher BMI, exercised less frequently, drank less frequently, and smoked more frequently than HC. Fewer blood samples were collected from fasting patients (Table 1).

**Table 1:** Demographics of the study population (*n* = 664).

Characteristics	Patients	Controls	Statistics	*p* value^a^
	(*n* = 504)	(*n* = 160)		
Age, mean (SD), years	36.42 (12.60)	35.94 (11.20)	*t* = 0.451	0.65
Sex (Male), *n* (%)	191 (37.9%)	48 (30.0%)	*χ*^2^ = 3.287	0.07
BMI, mean (SD), kg/m^2^	24.60 (4.50)	22.08 (2.72)	*t* = 8.594	**<0.001**
Blood collection time: AM, *n* (%)	184 (36.5%)	69 (43.1%)	*χ*^2^ = 2.255	0.13
Fasting time: at least 8 h, *n* (%)	114 (22.6%)	87 (54.4%)	*χ*^2^ = 58.022	**<0.001**
Alcohol drinking (at least once a week), *n* (%)	153 (30.4%)	75 (46.9%)	*χ*^2^ = 14.696	**<0.001**
Exercise (moderate), *n* (%)	173 (34.3%)	116 (72.5%)	*χ*^2^ = 72.001	**<0.001**
Current smoker, *n* (%)	152 (30.2%)	8 (5.0%)	*χ*^2^ = 42.029	**<0.001**

Categorical variables based on chi-squares tests and continuous variables based on *t*-tests.

*SD* standard distribution, *BMI* body mass index.

^a^Boldface values are statistically significant at *p* < 0.05.

### Selection of proteomic candidates that significantly differ between patients and HC

Of the 588 peptide markers, 133 peptides were initially excluded, as the PAR was ≤0.01 or ≥100, for at least 5% of the total study population. A total of 101 peptides were statistically significant in univariate analysis with the batch-corrected PAR values of the peptides as the dependent variable and patients versus HC as the independent variable. After additional control with age, sex, BMI, blood collection time, fasting time, alcohol use, exercise, and smoking behavior, only 30 peptides remained significant. Additional control of hospital type resulted in 18 peptides that were significantly different between patients and HC. Of the 18 peptides, six were excluded as they were significantly different between at least two hospitals in univariate analysis, when analyzed only in patients. The resulting 12 peptides were subjected to LCA (Supplementary Table 1 and Supplementary Fig 1).

### LCA

The fit indices of the LCA are summarized in Table 2. The Akaike information criteria decreased continuously as classes increased; however, the BIC was lowest with the two-class model, and the ssaBIC was lowest with the three-class model. The Lo–Mendell–Rubin likelihood ratio value was only significant for the two-class model, whereas bootstrapped likelihood ratio test was significant for all classes. Additionally, for models with three and four classes, the proportion of the smallest group was less than 50 individuals. In conclusion, the overall indices suggested that the two-class model was accepted as the final model. The latent class classification and protein expression tertiles are presented in Supplementary Table 2. Considering the differences in peptide levels, Class 1 was named the GELS-increased class, and Class 2 was named the GELS-decreased class.

**Table 2:** Comparisons of fit indices of latent class analysis (*n* = 504).

Model	AIC	BIC	ssaBIC	Entropy	LMR-LR *p* value	BLRT *p* value	*N* (proportion %)			
							1	2	3	4
1 class	13336.81	13438.16	13361.98	N/A	N/A	N/A	504 (100.0%)			
2 class	13187.46	13394.36	13238.83	0.604	0.0001	<0.0001	265 (52.6%)	239 (47.4%)		
3 class	13155.09	13467.56	13232.67	0.750	0.0690	<0.0001	273 (54.2%)	215 (42.7%)	16 (3.2%)	
4 class	13133.93	13551.97	13237.73	0.800	0.7630	0.0200	235 (46.6%)	216 (42.9%)	31 (6.2%)	22 (4.4%)

*AIC* Akaike information criteria, *BIC* Bayesian information criterion, *ssaBIC* sample-size adjusted Bayesian information criterion, *LMR-LR* Lo–Mendell-Rubin likelihood ratio, *BLRT* Bootstrapped likelihood ratio test, *N/A* not applicable.

No significant differences were found between the latent classes when the demographic characteristics were considered (Supplementary Table 3). Although significant differences were not observed in the total scores of clinician rater scales, the negative symptom factor of the BPRS was higher in the GELS-decreased latent class (*t* = −2.070, *p* = 0.039). Considering the individual items, the self-neglect and motor retardation items of the BPRS were increased in the GELS-decreased latent class, and the excitement and motor hyperactivity items of the BPRS were increased in the GELS-increased latent class (Table 3).

**Table 3:** Clinical characteristics between latent classes (*n* = 504).

Characteristics	Class 1 GELS-increased	Class 2 GELS-decreased	Statistics	*p* value^a^
	*n* = 239	*n* = 265		
Total scores of				
BPRS, mean (SD)	40.90 (9.52)	41.59 (9.18)	*t* = −0.810	0.42
YMRS, mean (SD)	4.29 (6.02)	3.65 (5.07)	*t* = 1.293	0.20
MADRS, mean (SD)	18.71 (11.18)	19.51 (11.29)	*t* = −0.796	0.43
HAM-A, mean (SD)	11.18 (7.05)	11.26 (7.19)	*t* = −0.133	0.90
Significant subscales/items				
BPRS negative symptoms factor, mean (SD)	4.24 (2.13)	4.65 (2.28)	*t* = −2.070	**0.039**
BPRS13, mean (SD) (self-neglect)	1.15 (0.52)	1.26 (0.66)	*t* = −2.235	**0.026**
BPRS18, mean (SD) (motor retardation)	1.36 (0.80)	1.52 (0.92)	*t* = −2.157	**0.031**
BPRS21, mean (SD) (excitement)	1.32 (0.81)	1.17 (0.52)	*t* = 2.418	**0.016**
BPRS23, mean (SD) (motor hyperactivity)	1.15 (0.44)	1.08 (0.30)	*t* = 1.981	**0.048**

Statistical analysis based on *t*-tests.

*BPRS* Brief Psychiatric Rating Scale, *YMRS* Young Mania Rating Scale, *MADRS* Montgomery–Asberg Depression Rating Scale, *HAM-A* Hamilton Anxiety Scale.

^a^Boldface values are statistically significant at *p* < 0.05.

Next, when including HC, two latent classes were identified. The proportion of HC and various total scores/factors/items of rating and subjective scales differed between classes. After additional adjustment for HC in a linear regression model, the negative symptom factor of the BPRS (*t* = −2.372, *p* = 0.018), the emotional withdrawal and motor retardation item of the BPRS, and the lassitude, inability to feel, and suicidal thoughts items of the MADRS were increased in a latent class when compared with the other class (Supplementary Tables 4,5).

### Bioinformatics analysis

**Fig. 1:**
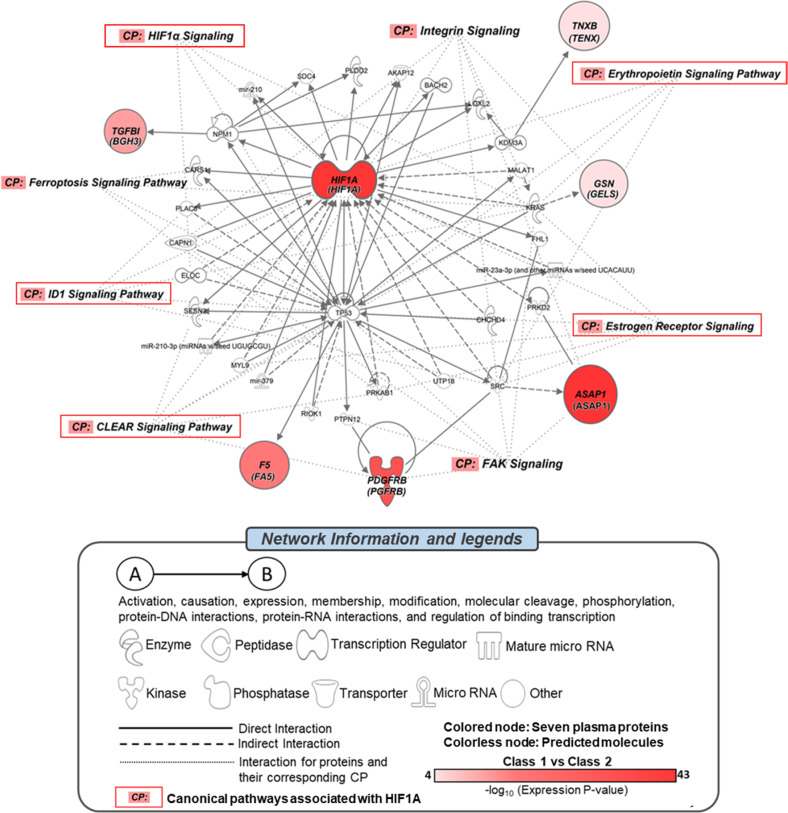
Protein network and associated canonical pathways generated by IPA for eight proteins differentiating latent classes. Seven of the eight proteins were included in the protein network. Direct and indirect interactions are represented by solid and dashed lines. Proteins are represented by a gene symbol with a protein entry name in parentheses. Shapes notify the molecular classes of proteins defined in the legend. Colored nodes notify the seven proteins, and white nodes represent predicted molecules. Canonical pathways involved in proteins in the network are represented by dotted lines. Canonical pathways in the red box are related to hub protein (HIF1A). Differences in protein expression levels of the seven features between the latent classes are represented by −log_10_ (expression *P* value). IPA ingenuity pathway analysis, CP canonical pathway.

Among the eight proteins that differed between clusters (Supplementary Table 2), seven [ASAP1 (ASAP1), TGFBI (BGH3), F5 (FA5), GSN (GELS), HIF1A (HIF1A), PDGFRB (PGFRB), and TNXB (TENX) (represented by the gene symbol)] were included in the network (network score = 18), which comprised 35 molecules. Through criteria of determination for diseases/functions and canonical pathways, the network was associated with a total of eight diseases/functions, namely, dermatological diseases and conditions, inflammatory disease, inflammatory response, immunological disease, neurological disease, nervous system development and function, cellular assembly and organization, and cellular function and maintenance (Supplementary Table 6). Furthermore, the network was related to eight canonical pathways: HIF1α, integrin, CLEAR, FAK, erythropoietin, estrogen receptor, ferroptosis, and ID1 signaling pathways (Fig. 1).

## Discussion

This study enabled us to differentiate a group of patients within the transdiagnostic psychosis-affective spectrum into two latent classes based on 12 pathological peripheral plasma markers. The two classes differed significantly among eight peptides, and negative symptoms were significantly different between these classes. This tendency was preserved when HC was included.

The multiplex platform for quantifying proteomes in plasma enabled us to identify significant proteins (peptides) that differed between patients and HC. The number of proteins quantified was larger than that in previous studies, and the study population number was sufficient to control for multiple covariates known to be associated with proteomic expression [21] when selecting pathological proteins (significantly different proteins between patients and HC). Although these pathological proteins do not always reflect pathophysiology within patients, as shown in our previous study [7], protein selection was performed to reduce the likelihood of reflecting factors other than psychopathology [5, 6]. Additionally, although multiple covariates were controlled for when selecting pathological proteins, stratification by sex before conducting LCA enabled us to discard the effects of sex-specific protein expression. This was in line with previous studies that required stratification by both age and sex [5, 6]. Moreover, the LCA results were similar when the data were reanalyzed and HC was included. The results indicate that the classes are not specific only to psychiatric disorders but also a continuum with the normal population.

The study revealed 12 proteins that differed between patients and HC. ASAP1, BGH3, FA5, GELS, PFGRB, TENX, and VWF were increased in patients, and FCGBP, HIF1A, PAFA, S10A2, and SEM6C were increased in HC. The most significant pathological protein was PFGRB. Elevated levels of PFGRB in the cerebrospinal fluid is seen as a marker of blood–brain barrier dysfunction [22]. Interestingly, there is evidence that the levels of PGFRB in the cerebrospinal fluid and serum are positively associated [23]. As these psychiatric disorders have evidence of a leaky blood–brain barrier [24], this could have been reflected in the plasma of the study patients. The increased level of circulating VWF in patients, is in line with previous studies of SCZ, BD, and MDD [25, 26]. This implies that these psychiatric diseases might share a common mechanism of endothelium-related inflammation. Interestingly, VWF itself is known to influence blood–brain barrier permeability [27]. Another protein that has been reported in multiple psychiatric disorders was HIF1A. This molecule was also a key protein in the network analysis. HIF1A has been proposed to have a protective effect on depression [28]. However, its mRNA expression within peripheral white blood cells seems to be state-dependent in MDD and BD [29]. Although further studies are needed, this does imply that these diseases could be commonly involved in pathways of energy metabolism and oxidative stress.

Recent studies have started to apply transdiagnostic approaches to cluster patients with psychosis-affective disorders, considering biological correlates. Stein et al. (2021) revealed that the negative syndrome in a transdiagnostic sample like ours is associated with the gray matter volume of the bilateral frontal opercula, and that no association exists between the diagnosis of the patients and the gray matter volume [30]. However, the study clustered the patients based on clinical data and analyzed the associations with gray matter volumes, showing multiple associations between other clinical clusters and other brain region volumes [30], which differed from our bottom-up approach using biological data to cluster patients. Another study by Chang et al. (2021) subtyped transdiagnostic patients based on frontal-posterior functional imbalance and found that the distribution of SCZ, BD, and MDD differs between clusters, but does not differ in clinical symptoms [31]. More studies should be conducted using various biological correlates with a data-driven approach to cluster these patients and investigate their associations with clinical traits and symptoms.

Consensus has been reached on negative symptom domains that include blunted affect, anhedonia, alogia, avolition, and asociality, and two factors, namely, amotivation and diminished expression [32]. Our study revealed that the items, including emotional withdrawal, motor retardation, lassitude, and inability to feel, were associated with latent classes when analyzing patients, or patients with HC. Evaluations using scales specific for negative symptoms are needed to confirm the associations between specific domains and factors. Even though efforts have expanded, the reports on the pathological mechanisms of negative symptoms are inconsistent, since negative symptoms are also heterogeneous in its nature [32]. Most studies have been based on patients with psychosis, in which several studies have shown significant associations between negative symptoms and inflammatory biomarkers [33].

The present study revealed that specific pathways, including systemic inflammation, hypoxia, and signal transduction, were associated with latent classes. Estrogen receptor, erythropoietin, and integrin pathways have been proposed as significant pathophysiologies in previous studies of SCZ, BD, and MDD, including a recent systematic review of peripheral blood proteomes [34–39]. However, as these pathways have associations with multiple psychiatric conditions, it could be associated with a common psychiatric dimension. The present study proposes that negative symptoms have a potential association with these pathways. Especially, the estrogen receptor pathway has been proposed for its ameliorative role in negative symptoms of SCZ not only due to sex differences in its severity and prognosis, but as hormonal replacement therapy has a protective effect for negative symptoms in women [40]. Additionally, a recent report of single-cell level lymphocytes revealed that NF-kB p65 and Stat 3 cell signaling alterations were shared between MDD and SCZ in a transdiagnostic sample, and suggested that they could represent a shared substrate for negative symptomology [41]. Both proteins are known to be associated with integrin pathways [42, 43]. However, cautious interpretation of specific results is necessary because the proteins were not from the CNS, and these pathways all have intracellular components. Even though the seven proteins of the network are known to have secretory pathways or have the potential to be secreted from intracellular to extracellular regions [44–48], the link between plasma and the CNS is still under investigation. Nevertheless, considering that these symptoms are resistant to treatment, investigating the proposed mechanisms could expand our knowledge of their pathophysiology.

By contrast, the differential diagnosis of SCZ, BD, and MDD were not associated with latent classes. This implies that without the consideration of negative symptoms, the conventional differentiation between SCZ, BD, and MDD based on proteomics will have limitations, as it does not reflect systemic biological manifestations. Therefore, phenotypes should consider not only the ICD (International Statistical Classification of Diseases and Related Health Problems) or DSM (Diagnostic and Statistical Manual of Mental Disorders)-based symptom checklists but also the relationship between negative symptoms and associated biological correlates. This will deepen our understanding of the pathophysiology of psychotic-affective disorders and enable us to explain the heterogeneity within and the overlap between disorders.

However, the results also imply the obvious gap between circulating proteins and psychiatric manifestations, including structurally, the blood–brain barrier. These gaps are probably why a clearer separation in psychiatric manifestations between the latent classes were not seen, even though we selected proteins that differentiated psychiatric diseases and HC, and controlled significant covariates. There could be other biological measures that are better for biological subtyping [5], that might reflect psychiatric symptoms with a stronger association.

### Strengths and limitations

This study has the following limitations. First, it was a cross-sectional study; therefore, causality could not be determined. A longitudinal study with multiple measurements of clinical symptoms and plasma proteomes would enable us to investigate the preservation of latent classes. Second, the proteins were obtained from the blood; therefore, functional analysis has limitations. Although there is evidence of blood–brain barrier dysfunction in psychiatric disorders, blood does not always reflect the CNS. Third, the differences between the scales were small. The scales themselves might not be able to capture negative symptoms sensitively; therefore, more detailed evaluations should be performed in the future. Especially the results of the individual items need to be cautiously interpreted, as they are numerically very close. Fourth, biological analysis was conducted using proteomics only. Integration with other omics or other biological correlates could capture sophisticated mechanisms that would reveal more accurate biological subtypes. Fifth, there were several preprocessing procedures to conduct LCA, including stratification by sex, and decreasing the dimension of protein values from a continuous to a discrete variable [6], which could have resulted in the loss of information, and affect the latent classes. However, LCA has its advantages, as it is considered a more statistically robust method than cluster analysis, since it is model-based, and generates fit statistics [49]. Finally, independent validation was not performed in this study.

Nevertheless, the strength of the study was that it was the first, to the knowledge of the authors, to compare a transdiagnostic psychosis-affective disorder population with numerous proteomic targets from the blood. Multiplexing proteins simultaneously enabled us to select significant pathological proteins and cluster patients into two biological subgroups. Negative symptoms need more attention because they vary between these subgroups, which tend to be neglected and remain poorly understood. Further biological studies should use longitudinal designs with detailed evaluation of negative symptoms to deepen our understanding of psychosis-affective disorders.

## Supplementary information

Supplementary methods

Supplementary Figure

Supplementary table 1

Supplementary table 2

Supplementary table 3

Supplementary table 4

Supplementary table 5

Supplementary table 6

## Data Availability

The quantitation information of 12 targets included in the latent classes were deposited to the Panorama Public repository (https://panoramaweb.org/Zzgv9f.url). Email: panorama+reviewer162@proteinms.net, Password: TOUvWkEL. The other datasets presented in this study may be available from the corresponding authors on reasonable request.
