# Supplementary material for: Latent class analysis of psychotic-affective disorders with data-driven plasma proteomics
Source: Transl Psychiatry. 2023 Feb 6;13:44. doi: 10.1038/s41398-023-02321-9 (PMC9902608; doi:10.1038/s41398-023-02321-9)
Supplement: Supplementary file 1 — Supplementary methods [file 41398_2023_2321_MOESM1_ESM.docx]

**Supplementary Methods**

**Latent class analysis of psychotic-affective disorders with data-driven plasma proteomics**

Sang Jin Rhee^1,#^, Dongyoon Shin^2,#^, Daun Shin^3,4^, Yoojin Song^3,4^, Eun-Jeong Joo^5,6^, Hee Yeon Jung^3,7,8^ Sungwon Roh^9^, Sang-Hyuk Lee^10^, Hyeyoung Kim^11^, Minji Bang^10^, Kyu Young Lee^5,12^, Se Hyun Kim^4^, Minah Kim^3,4^, Jihyeon Lee^2^, Jaenyeon Kim^2,13^, Yeongshin Kim^2^, Jun Soo Kwon^3,4,8^, Kyooseob Ha^3,4,8^, and Youngsoo Kim^2,13*^, Yong Min Ahn^3,4,8*^

Running title: Plasma proteome-based clustering of psychotic-affective disorders

^1^Biomedical Research Institute, Seoul National University Hospital, Seoul, Republic of Korea. ^2^Department of Biomedical Sciences, Seoul National University College of Medicine, Seoul, Republic of Korea. ^3^Department of Psychiatry, Seoul National University College of Medicine, Seoul, Republic of Korea. ^4^Department of Neuropsychiatry, Seoul National University Hospital, Seoul, Republic of Korea. ^5^Department of Neuropsychiatry, School of Medicine, Eulji University, Daejeon, Republic of Korea. ^6^Department of Psychiatry, Uijeongbu Eulji Medical Center, Eulji University, Uijeongbu, Republic of Korea. ^7^Department of Psychiatry, SMG-SNU Boramae Medical Center, Seoul, Republic of Korea. ^8^Institute of Human Behavioral Medicine, Seoul National University Medical Research Center, Seoul, Republic of Korea. ^9^Department of Psychiatry, Hanyang University Hospital and Hanyang University College of Medicine, Seoul, Republic of Korea. ^10^Department of Psychiatry, CHA Bundang Medical Center, CHA University School of Medicine, Seongnam, Republic of Korea. ^11^Department of Psychiatry, Inha University Hospital, Incheon, Republic of Korea. ^12^Department of Psychiatry, Nowon Eulji University Hospital, Seoul, Republic of Korea. ^13^Institute of Medical and Biological Engineering Medical Research Center, Seoul National University College of Medicine, Seoul, Republic of Korea

**Plasma sample preparation for LC-MRM-MS analysis**

For the targeted proteomic analysis, plasma samples were thawed on ice and centrifuged at 10,000 *g* for 10 min at 4°C. Supernatants were transferred to new tubes and vortexed. For each sample, 44 μL was diluted 1:4 with MARS buffer A (Agilent Technologies, Santa Clara, CA, USA) and passed through 0.22-µm Spin-X filters (Corning Costar, NY, USA). A volume of 176 μL of buffer A was added to each sample, and each diluted sample was centrifuged through a 0.22-μm filter (12,000 *g*, room temperature). Each plasma sample was depleted of 6 high-abundance human plasma proteins (albumin, IgG, IgA, transferrin, haptoglobin, and antitrypsin) using a multiple affinity removal system (MARS) column (Hu-6HC, 4.6 × 100 mm, Agilent Technologies, Santa Clara, CA, USA) that was loaded onto a high-performance liquid chromatography (HPLC) system (Shimadzu Co, Kyoto, Japan). A total of 200 μL was injected for each sample.

Depleted plasma samples were concentrated by centrifugal filtration for 6 hours at 4°C using a 3000-Da molecular weight cutoff (MWCO) filter (Amicon Ultra-4 3K, Millipore, Burlington, MA, USA). The concentrated proteins of individual samples were then quantified by bicinchoninic acid assay (BCA assay) using the Pierce™ BCA Protein Assay Kit (Thermo Scientific, Rockford, IL, USA). A 6-point standard curve was generated by serially diluting an initial concentration of 2 mg/mL BSA by a factor of 2. Standards and samples were placed on a 96-well plate, and a mixture of copper solution and BCA solution (1:50) was added.

The proteins were digested with RapiGest surfactant and trypsin. Next, 40 μL 0.2% RapiGest, 20 mM dithiothreitol (DTT), and 100 mM ABC buffer, pH 8.0 was added to the 40-μl plasma samples, adjusted with HPLC-grade water to a 100-μg digestion. After 1 hour in 60°C, 20 μL 100 mM iodoacetamide (IAA) was added, and the samples were incubated in the dark for 30 min at room temperature. Next, the samples were incubated for 4 hours at 37°C after trypsin, dissolved in 50 mM ABC, pH 8.0, was added (sequencing-grade modified, Promega, Madison, WI, USA). Then, 10% formic acid was added to the samples to 1% to stop the enzymatic reaction and incubated for 30 min at 37° to hydrolyze RapiGest surfactant in the acidified samples. After centrifugation at 15,000 rpm at 4°C for 1 hour, the cleaved RapiGest surfactant precipitated, and the supernatant was transferred to a clean tube.

The plasma peptide samples (the transferred supernatant for each sample) were spiked with crude stable isotope-labeled internal standard (SIS) peptide (purity >70%), in which a C-terminal lysine or arginine was heavy-isotope-labeled (^13^C_6_^15^N_2_ or ^13^C_6_^15^N_4_) (JPT, Berlin, Germany). A total of 675 plasma samples were distributed among 5 preparation batches (batches 1–5), because they were collected at various time points. In each preparation batch, the samples were randomly distributed and assigned identification numbers to blind the researchers throughout the sample preparation.

**Determination of quantifiable targets for LC-MRM-MS analysis**

For the targeted proteomic analysis, integrated protein targets for MDD, BD, SCZ, and HC were selected. Three sources were compiled to generate the list of integrated candidate targets: 1) new targets for psychiatric disorders (MDD, BD, and SCZ), 2) established targets for mood disorders (MDD and BD), and 3) laboratory-established targets.

New candidates for psychiatric disorders were collected from 5 databases on psychiatric disorders (MDD, BD, and SCZ): PsyGeNET (http://www.psygenet.org), Schizophrenia Gene Resource 2 (SZGR2) (https://bioinfo.uth.edu/SZGR/), Laboratory of Neurophenomics (http://www.neurophenomics.info/), Comprehensive Database for Schizophrenia (SZDB2) (www.SPRdb.org), and The Stanley Neuropathology Consortium Integrative Database (SNCID) (http://sncid.stanleyresearch.org) [1-4]. As a result, 8081 genes were integrated as initial targets. The Human Blood Proteins Atlas and Plasma Proteome Database (PPD) were used to filter and select targets that are detectable in blood [5, 6]. In total, 1462 blood-detectable proteins were selected.

To examine targets that had matching MS/MS spectra and unique peptides, 8 MS/MS spectral libraries from various institutes—the Institute for Systems Biology (https://www.systemsbiology.org), National Institute of Standards and Technology (https://www.nist.gov), and the SWATHAtlas database (www. SWATHAtlas.org)—were used. In total, 407 proteins, corresponding to 407 unique peptides with top 10 transitions per each peptide, were selected. The established candidates of mood disorders (ie, MDD and BD) were drawn from our previous study [7]. The laboratory-established targets included proteins that have been approved by the US Food and Drug Administration (FDA) and designated as laboratory developed tests (LDTs) and proteins that have been developed in our previous research, unrelated to psychiatric disorders. In total, 1667 proteins/2283 peptides were merged as integrated candidate targets.

To examine targets that were detected and quantified in blood samples of psychiatric disorders, LC-MRM-MS analysis was performed on a pooled plasma sample that consisted of 50 HCs, 50 MDD, 50 BD, and 50 SCZ samples. Targets were considered to be detectable and quantifiable if: 1) at least 5 transitions for LC-MRM-MS were observed; 2) they had the same elution patterns within the predicted retention time (RT) (±5 min); 3) the ratio of transition peaks was obtained as in the spectral library (dot product ≥ 0.6); 4) RTs and dot products were equal between light and heavy peptides; and 5) there were transitions that had the highest intensity based on the rank of intensity and that were filtered by AuDIT for selecting interference-free transitions [8].

Regarding the 2283 unique peptides that corresponded to 1667 proteins and the relevant 2283 stable isotope-labeled internal standard (SIS) peptides, LC-MRM-MS analysis was performed to measure the detectability of the integrated candidate targets. A total of 642 target peptides were selected as being quantifiable.

**LC-MRM-MS analysis**

For our targeted proteomic analysis, target peptides were analyzed by targeted LC-MRM-MS on an Agilent 6490 triple quadrupole (QQQ) mass spectrometer (Agilent Technologies, Santa Clara, CA, USA) that was equipped with a Jetstream electrospray source that was coupled to a 1260 Infinity HPLC system (Agilent Technologies, Santa Clara, CA, USA). Solvents A and B for the HPLC consisted of 0.1% formic acid/water (v/v) and 0.1% formic acid/acetonitrile (v/v), respectively. Glass vials of the samples in the autosampler were maintained at 4°C.

A total of 40 μl of digested sample was injected into a guard column (2.1 × 15.0 mm, 1.8 µm, 80 Å) (Agilent Technologies, Santa Clara, CA, USA). Online desalting was conducted with the effluent toward waste at 50 μl/min for 10 min in 3% solvent B, consisting of 0.1% formic acid/acetonitrile (v/v), at 40°C. After the position of valve was switched, the desalted sample was transferred to the analytical column (0.5 × 35.0 mm, 3.5 µm, 80 Å) (Agilent Technologies, Santa Clara, CA, USA) in 3% solvent B at a flow rate of 40 µL/min for 5 min. The analytical column was heated and maintained at 40°C by an oven.

The total run time per LC-MRM-MS analysis was 70 min. Approximately 10 μg of digested peptides was injected per LC-MRM-MS run. The peptides were separated on the column and eluted with a linear gradient of 3% to 35% acetonitrile (ACN) with 0.1% formic acid (FA) for 50 min at 40 µL/min. The mass spectra were generated in positive ion mode, based on the following parameters: 2500 V ion spray capillary voltage, 2000 V nozzle voltage, 5 V cell accelerator voltage, 200 V delta EMV, and 380 V fragmented voltage. The drying gas was sprayed at 15 L/min at 250°C, and the sheath gas flow was 12 L/min at 350°C. Collision energy (CE) was optimized by adding the intensities of individual transitions that resulted in the largest peak area. The default value for CE was calculated as follows: CE = 0.031 × (m/z of precursor) + 1 for doubly charged precursor ions and CE = 0.036 × (m/z of precursor) – 4.8 for triply charged ions. Five additional steps of adding or subtracting 2 V on either side of the default CE value were predicted for determining the optimized CE.

Before the analysis of individual plasma samples, SIS peptides that corresponded to the determined targets were pooled and analyzed to evaluate their RTs. The RTs of the SIS peptides were compared with those of endogenous target peptides by spiking the pooled mixture of SIS peptides (10, 100, or 1000 fmol of heavy peptides corresponding to each target) with 100 fmol of a heavy peptide of beta-galactosidase (β-gal), which was used to address technical variations across LC-MRM-MS runs and between preparations by different researchers. Subsequently, the final targets were quantified in individual blood samples, which were listed randomly in blocked batches with an identification number for each sample. LC-MRM-MS analysis was performed once per sample (1 replicate for each sample).

**Processing of LC-MRM-MS data**

The raw data from the LC-MRM-MS analysis were processed in Skyline (version 19.1.0) (MacCoss Lab, Seattle, WA, USA) to calculate the peak area values of the transitions. Peptide-transition peaks were investigated using peak integrations that were performed manually. Peptide quantification was based on the relative abundance of the endogenous and SIS peptide transitions—the relative abundance of the transition pairs (Q1 and Q3) was determined by the ratio of endogenous (Light) to SIS (Heavy) peptide peak areas, reported as the peak area ratio (PAR), or Light/Heavy (L/H) ratio.

After the manual peak integrations, unstable targets were examined across individual samples. A total of 54 unstable targets with low intensity (intensity < 1000), unequal RTs between light and heavy peptide, and skewed peaks were excluded. Subsequently, PAR values of 588 targets across 675 individual samples were generated and normalized by the area of heavy β-gal peptide to reduce the technical variability from the sample preparations between researchers.

**References**

1. Gutierrez-Sacristan A, Grosdidier S, Valverde O, Torrens M, Bravo A, Pinero J, et al. PsyGeNET: a knowledge platform on psychiatric disorders and their genes. Bioinformatics. 2015;31(18):3075-7.

2. Jia P, Han G, Zhao J, Lu P, Zhao Z. SZGR 2.0: a one-stop shop of schizophrenia candidate genes. Nucleic Acids Res. 2017;45(D1):D915-D24.

3. Wu Y, Li X, Liu J, Luo XJ, Yao YG. SZDB2.0: an updated comprehensive resource for schizophrenia research. Hum Genet. 2020;139(10):1285-97.

4. Kim S, Webster MJ. The Stanley Neuropathology Consortium Integrative Database (SNCID) for Psychiatric Disorders. Neurosci Bull. 2019;35(2):277-82.

5. Nanjappa V, Thomas JK, Marimuthu A, Muthusamy B, Radhakrishnan A, Sharma R, et al. Plasma Proteome Database as a resource for proteomics research: 2014 update. Nucleic Acids Res. 2014;42(Database issue):D959-65.

6. Ponten F, Schwenk JM, Asplund A, Edqvist PH. The Human Protein Atlas as a proteomic resource for biomarker discovery. J Intern Med. 2011;270(5):428-46.

7. Shin D, Rhee SJ, Lee J, Yeo I, Do M, Joo EJ, et al. Quantitative Proteomic Approach for Discriminating Major Depressive Disorder and Bipolar Disorder by Multiple Reaction Monitoring-Mass Spectrometry. J Proteome Res. 2021;20(6):3188-203.

8. Abbatiello SE, Mani DR, Keshishian H, Carr SA. Automated detection of inaccurate and imprecise transitions in peptide quantification by multiple reaction monitoring mass spectrometry. Clin Chem. 2010;56(2):291-305.
