# Supplementary material for: Latent class analysis of psychotic-affective disorders with data-driven plasma proteomics
Source: Transl Psychiatry. 2023 Feb 6;13:44. doi: 10.1038/s41398-023-02321-9 (PMC9902608; doi:10.1038/s41398-023-02321-9)
Supplement: Supplementary file 2 — Supplementary Figure [file 41398_2023_2321_MOESM2_ESM.pptx]

## Slide 1
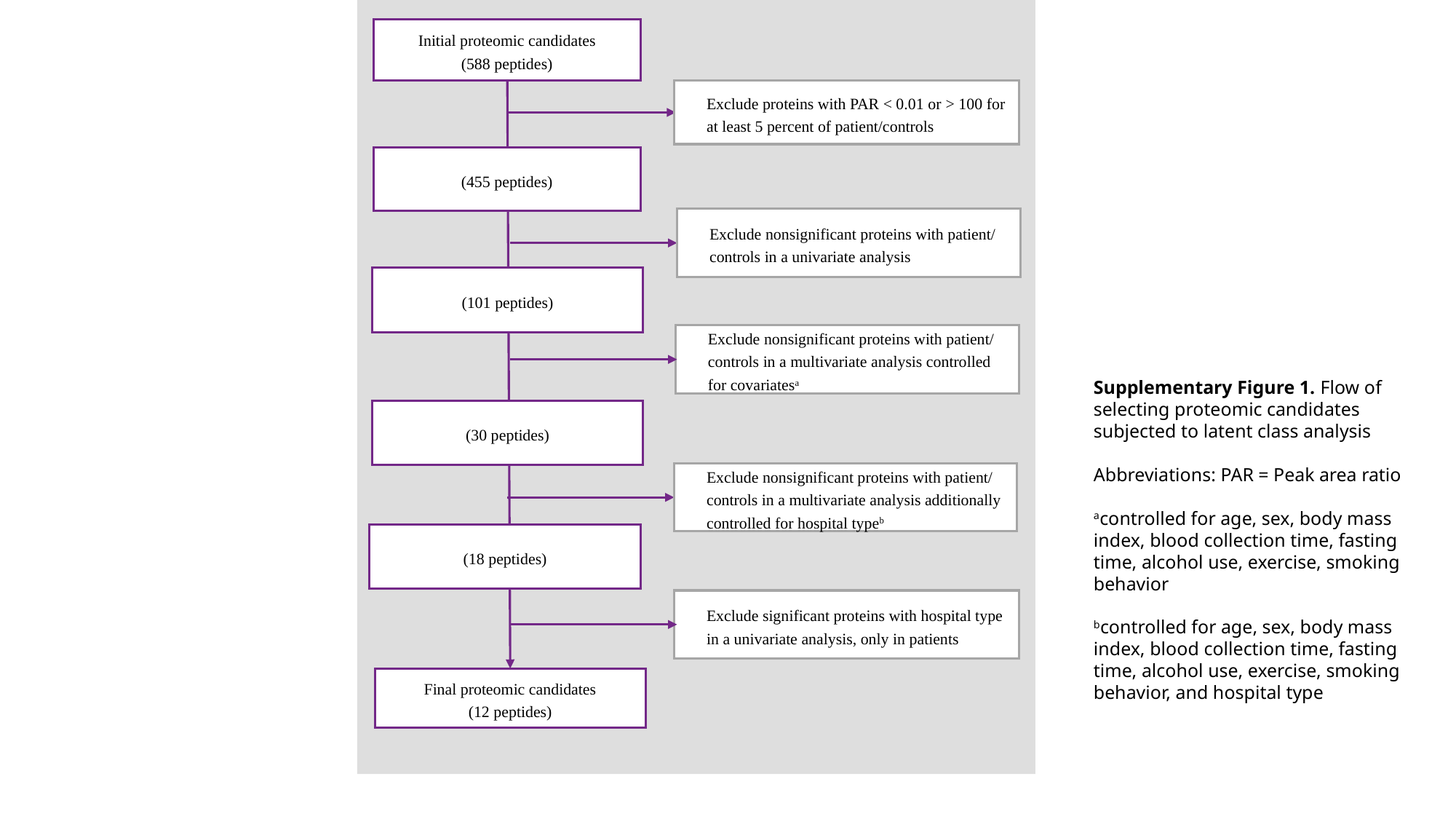

Initial proteomic candidates
(588 peptides)
Exclude proteins with PAR < 0.01 or > 100 for at least 5 percent of patient/controls
(101 peptides)
(455 peptides)
Exclude nonsignificant proteins with patient/controls in a univariate analysis
Exclude nonsignificant proteins with patient/controls in a multivariate analysis controlled for covariatesa
Supplementary Figure 1. Flow of selecting proteomic candidates subjected to latent class analysis
Abbreviations: PAR = Peak area ratio
acontrolled for age, sex, body mass index, blood collection time, fasting time, alcohol use, exercise, smoking behavior
bcontrolled for age, sex, body mass index, blood collection time, fasting time, alcohol use, exercise, smoking behavior, and hospital type
(30 peptides)
Exclude nonsignificant proteins with patient/controls in a multivariate analysis additionally controlled for hospital typeb
(18 peptides)
Exclude significant proteins with hospital type in a univariate analysis, only in patients
Final proteomic candidates
(12 peptides)
